# Development of *Cissus quadrangularis*-Loaded POSS-Reinforced Chitosan-Based Bilayer Sponges for Wound Healing Applications: Drug Release and *In Vitro* Bioactivity

**DOI:** 10.1021/acsomega.3c00999

**Published:** 2023-05-23

**Authors:** Sibel Deger Aker, Sedef Tamburaci, Funda Tihminlioglu

**Affiliations:** Department of Chemical Engineering, Izmir Institute of Technology, Gulbahçe Campus, Urla, İzmir 35430, Turkey

## Abstract

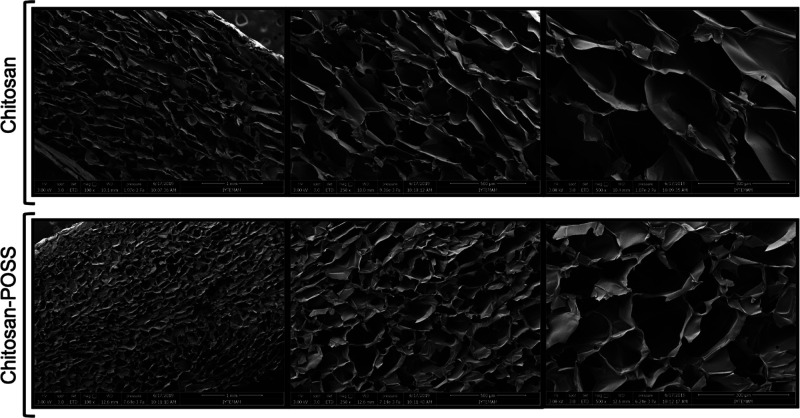

Nowadays, antibiotic-loaded biomaterials have been widely used in wound healing applications. However, the use of natural extracts has come into prominence as an alternative to these antimicrobial agents in the recent period. Among natural sources, *Cissus quadrangularis* (CQ) herbal extract is used for treatment of bone and skin diseases in ayurvedic medicine due to its antibacterial and anti-inflammatory effects. In this study, chitosan-based bilayer wound dressings were fabricated with electrospinning and freeze-drying techniques. CQ extract-loaded chitosan nanofibers were coated on chitosan/POSS nanocomposite sponges using an electrospinning method. The bilayer sponge is designed to treat exudate wounds while mimicking the layered structure of skin tissue. Bilayer wound dressings were investigated with regard to the morphology and physical and mechanical properties. In addition, CQ release from bilayer wound dressings and *in vitro* bioactivity studies were performed to determine the effect of POSS nanoparticles and CQ extract loading on NIH/3T3 and HS2 cells. The morphology of nanofibers was investigated with SEM analysis. Physical characteristics of bilayer wound dressings were determined with FT-IR analysis, swelling study, open porosity determination, and mechanical test. The antimicrobial activity of CQ extract released from bilayer sponges was investigated with a disc diffusion method. Bilayer wound dressings’ *in vitro* bioactivity was examined using cytotoxicity determination, wound healing assay, proliferation, and the secretion of biomarkers for skin tissue regeneration. The nanofiber layer diameter was obtained in the range of 77.9–97.4 nm. The water vapor permeability of the bilayer dressing was obtained as 4021 to 4609 g/m^2^day, as it is in the ideal range for wound repair. The release of the CQ extract over 4 days reached 78–80% cumulative release. The release media were found to be antibacterial against Gram-negative and Gram-positive bacteria. *In vitro* studies showed that both CQ extract and POSS incorporation induced cell proliferation as well as wound healing activity and collagen deposition. As a result, CQ-loaded bilayer CHI-POSS nanocomposites were found as a potential candidate for wound healing applications.

## 1 Introduction

Clinical methods such as skin transplantation are used to regenerate the skin tissue for wound healing. However, clinical methods have disadvantages such as the long duration of treatment, delayed healing in the wound area, and the risk of transmission of infectious diseases. To avoid these disadvantages, wound dressings, which are made up of synthetic and natural polymers, have been used according to the wound type.^1,2^ For wound healing applications, ideal wound dressings should meet major requirements such as biocompatibility, biodegradability, and suitable elasticity to mimic the skin tissue. In addition, it should prevent the wound area from microbial contamination.^3−5^ Natural polymers are preferred as wound dressings because of their structural similarity to the extracellular matrix (ECM) in skin tissue. This structural similarity of natural polymer matrices stimulates the cell bioactivity around the wound area, so it is more preferred than synthetic polymers.^6^ Chitosan as a natural polymer possesses ideal characteristics such as biodegradability, biocompatibility, nontoxicity, and antimicrobial activity.

In addition, chitosan supports granulation tissue formation in wound healing and blood clotting.^7^ However, chitosan has low stability and weak mechanical properties. To overcome this problem, generally, it is used as a polyelectrolyte complex constituted with anionic polymers such as alginate, gelatin, or a crosslinker (e.g., glutaraldehyde and genipin), or composite structures are formed using nanofillers (e.g., silica, clay, and polyhedral oligomeric silsesquioxane (POSS)) to increase the stability and mechanical property for wound dressing applications. Silicon (Si) as a significant element has an essential biological role in different tissues of the human body. Si is found in the connective tissues (bone, blood vessels, and skin) at high levels and has a role in bioactive reaction with skin tissue. This makes silica-based biomaterials come into prominence for skin regeneration.^8^ Si released from biomaterial can be found as organosilicons in the human body and can be metabolized to orthosilicic acid. Thus, Si can be transported through the basal membrane and reach the epidermal and dermal fibroblast cells. This may lead to inducing of the upregulation of fibroblast growth factor (β-FGF) expression.^9^

Among nanosilica sources, polyhedral oligomeric silsesquioxanes (POSS) are known as the smallest silica particles (1.5 nm) and hybrid (inorganic/organic) well-defined cage structures, which are comprised of a silicon/oxygen cage and hydrocarbon functional groups attached to corner Si molecules.^10^ In the literature, it is indicated that the use of POSS nanoparticles both enhance mechanical properties and stability and support wound healing.^11,12^

Recently, bioactive extract-loaded multilayered biomaterials have been designed as wound dressings to support wound repair by mimicking the morphology and structure of skin tissue layers, the epidermis and dermis, as well as showing antimicrobial activity and inducing wound healing.^13−16^ There are limited studies in the literature related to chitosan-based bilayer dressings.^17,18^ In addition to the wound dressing designs, recently, bioactive agents such as antimicrobial drugs and herbal extracts have been incorporated in wound dressings to accelerate wound healing and induce tissue formation. One of these herbal extracts is *Cissus quadrangularis* (CQ).^19^ CQ extract incorporation promotes wound healing due to its bioactive ingredients such as quercetin, ascorbic acid, vitamin C, and phenols.^20^ CQ extracts have been used commonly as bioactive agents for bone tissue regeneration.^21^ They are also known to contribute to the blood clotting effect and wound healing in Ayurveda.^22^ CQ has been used in wound healing as a crude extract only in traditional medicine.^20,23^ The use of direct extract may cause toxic or side effects. Therefore, the extract can be loaded into the polymer systems and sustained extract release can be observed for better wound healing effect.

The main objective of this study is to design a bilayer wound dressing composed of a CHI-CQ nanofiber layer and CHI-POSS porous layer to mimic the layered structure of the skin tissue as well as provide controlled release of antibacterial CQ extract with high wound healing capacity and induce the ECM production of fibroblasts with nanosilica POSS incorporation to the porous layer. Bilayer wound dressings were obtained by coating CQ-loaded nanofibers onto nanocomposite sponges with an electrospinning method. Bilayer wound dressings were characterized with regard to the physicochemical properties, *in vitro* extract release kinetics, and antimicrobial activity. The *in vitro* bioactivity of fibroblast and keratinocyte cells on each layer was investigated with cytotoxicity determination, wound healing assay, proliferation assays, and determination of biomarkers for skin tissue regeneration.

## 2 Materials and Methods

### 2.1 Materials

Medium molecular weight chitosan (Sigma-Aldrich; deacetylation degree, 77.5%) and low molecular weight chitosan (CHI) (Sigma-Aldrich; deacetylation degree, 75%) were used for nanofiber and sponge production, respectively. Acetic acid (Merck) was used as a solvent for polymer solution preparation. Polyhedral oligomeric silsesquioxane (POSS) (Hybrid Plastics) was used as a nanofiller for preparation of the bottom layer of wound dressing material. The upper layer was made of chitosan nanofiber consisting of *C. quadrangularis* (CQ with 3% ketosteroid) (Ambe Phytoextracts Pvt.) selected as an antimicrobial and bioactive extract for wound healing. Phosphate buffer saline (PBS) tablets (Invitrogen, Thermo Fisher Scientific) were used for the *in vitro* extract release study and swelling studies. Dulbecco’s modified Eagle medium (DMEM, Serox), fetal bovine serum (FBS, Serox), and penicillin and streptomycin antibiotic solution (Serox) were used for cell culture studies. The WST-1 assay (Biovision) was used for the *in vitro* cytotoxicity test.

### 2.2 Preparation of Bilayer Wound Dressing

#### 2.2.1 Extract-Loaded Nanofiber Production (Upper Layer)

Medium molecular weight polymer solution (2% (w/v)) was prepared by dissolving chitosan in 70% (v/v) acetic acid solution for 24 h using a magnetic stirrer. Then, 0.5, 1, 2, and 3% (w/w CHI) genipin as a natural crosslinker agent and polyethylene oxide (PEO) (CHI:PEO, 80:20) as a plasticizer were added to chitosan solution to obtain stable and homogeneous fiber morphology. Then, the *C. quadrangularis* (CQ) extract was used at three different concentrations with regard to the polymer/extract ratio (P:E of 2.5:1, 5:1, and 7.5:1). CQ powder was dispersed in 2 mL of distilled water, then added dropwise in genipin-crosslinked chitosan solution, and mixed for 1 h. The final solution was fabricated as extract-loaded nanofibers with an electrospinning system. CQ-loaded nanofibers were fabricated under the process conditions of 10 cm distance, 1.5 mL/h flow rate, and 20 kV voltage.^24^

#### 2.2.2 Fabrication of CHI-POSS Sponge (Bottom Layer)

Chitosan solution was prepared by dissolving 2% (w/v) low molecular weight chitosan (CHI) in 2% (v/v) acetic acid solution and stirring overnight. Then, POSS nanoparticles (CHI:POSS, 95:5) were dispersed in 5 mL of distilled water and added to homogeneous chitosan solution, which was mixed for 1 h. CHI-POSS solution was ultrasonicated for 30 min by using a Misonix Ultrasonicator to disperse POSS nanoparticles in the chitosan matrix effectively. Finally, CHI-POSS solution was poured into a 5 cm Petri dish and 12-well plates and incubated at −20 °C for 24 h for pre-freezing. Finally, the samples were lyophilized in a freeze dryer (Labconco, FreeZone 4.5 L Freeze Dry Systems, 77500-77510 Series Models) for 48 h at −46 °C and 0.018 mbar vacuum.^24^

#### 2.2.3 Fabrication of Bilayer Wound Dressing

Bilayer wound dressing was fabricated by coating CQ-loaded nanofibers (upper layer) on top of the CHI-POSS sponge layer (bottom layer) with the electrospinning method. Effects of the CHI:CQ ratio and genipin crosslinker concentration on the properties of wound dressing materials were investigated.^24^

### 2.3 Characterization of Bilayer Sponges

#### 2.3.1 Scanning Electron Microscopy (SEM) Analysis

SEM analysis (Quanta FEG 250, 7 × 10^–2^ mbar and 15 mA) was performed to investigate the morphology of the upper layer, nanofibers, as well as the bottom layer, sponge composites. In addition, the structural integrity of the bilayer composite and the thickness of each layer were observed with SEM. Samples were coated with gold in the presence of argon gas using an Emitech K550X Spot Coater before analysis. ImageJ software was used for quantitative determination of average nanofiber diameters.

#### 2.3.2 Fourier Transform Infrared Spectroscopy (FT-IR)

Characteristic peaks of CQ extract and POSS nanoparticles and chemical interaction between chitosan and CQ and that between chitosan and POSS were investigated with an ATR instrument (Shimadzu FTIR-8400s) at a wavelength range from 4000 to 400 cm^–1^ with 4 cm^–1^ resolution.

#### 2.3.3 Water Uptake Capacity

The water uptake capacity of each layer and bilayer wound dressings was determined with the swelling test. Before test, dry samples (*W*_d_) were weighed. Then, the samples were incubated in 1× PBS solution at 37 °C to mimic the human body condition. Finally, wet samples were taken from 1× PBS solution and weighed (*W*_w_) for 4, 24, and 48 h intervals. Swelling % was calculated according to eq 1.
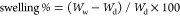
1

#### 2.3.4 Determination of Open Porosity with the Liquid Displacement Method

Open porosity % of single-layer and bilayer wound dressings was calculated with the liquid displacement method where ethanol was used as a liquid phase. Samples were put into a graduated cylinder containing 20 mL of ethanol (*V*_1_) and incubated in the vacuum oven for 1–2 min to remove air trapped in the porous structure. Ethanol entered when air exited the structure. The total volume of both ethanol and samples was measured as the *V*_2_ value. Then, samples were removed from the graduated cylinder containing ethanol and recorded as *V*_3_. Open porosity was calculated according to eq 2.
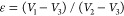
2

#### 2.3.5 Water Vapor Permeability

A two-chamber operating system was used to measure the water vapor permeability of CHI-POSS sponges. To maintain a relative humidity of 100% in the bottom chamber, a tiny container filled with deionized water was included. A moisture probe is located on the top chamber and is linked to the Datalogger SK-L 200 TH system. The humidity probe at the top records the relative humidity and temperature. Sponges were cut in a diameter of 4 cm and placed between two compartments. The relative humidity in the upper chamber was reduced to 5% with the anhydrous CaSO_4_ column before the test. Then, water vapor was allowed from the bottom to the sponge. Data were recorded to the computer at 1 min intervals via Datalogger. Water vapor permeability (WVP) is calculated with eq 3.
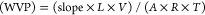
3

The slope is calculated from the time graph with [(*P*_IL_ – *P*_1ui_)/(*P*_IL_ – *P*_1ut_)]. *P*_1L_ and *P*_1u_ are partial pressures in the upper and lower chambers, respectively. *A* is the transfer area of the exposed film surface (m^2^), *t* is the test time, *R* is a gas constant, and *V* is the volume of the chamber. The WVP of chitosan-POSS sponges was calculated in mol/min cm kPa units. Water vapor transmission rates (WVTR) were calculated as g/m^2^ day.

#### 2.3.6 Mechanical Testing

Mechanical properties of CHI-POSS sponges (bottom layer) were determined with the tension test according to the ASTM D882 standard using a TAXT Plus Texture Analyzer (Stable Micro Systems, UK). Before the tension test, CHI-POSS sponges were cut into a rectangular shape (1 cm × 6 cm) and conditioned in a humidity chamber at 25 °C and 55% relative humidity conditions for 24 h. Digital calipers (Mitutoyo) were used to measure the thickness of CHI-POSS sponges. The tensile test was carried out using a 5 kgf load cell that has tensile grips at 10 mm min^–1^ crosshead speed. The tensile strength and Young’s modulus of samples were calculated for each sample.

#### 2.3.7 Enzymatic Degradation

Nanofiber-coated CHI-POSS sponges were incubated in enzymatic degradation solution as 1× PBS solution containing 1.5 μg/mL lysozyme and sodium azide (0.01%) at 37 °C. The lysozyme concentration was determined to obtain the concentration in serum to mimic the body microenvironment.^25^ Sodium azide was used in enzymatic solution to prevent bacterial contamination during incubation. Degradation solution was refreshed every other day to sustain enzymatic activity. Samples were weighed before experiment (*W*_0_). Then, samples were incubated in degradation solution and taken off to be weighed (*W*_1_) on 3rd, 7th, and 14th days to determine the weight change. Weight loss % was calculated using eq 4.
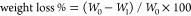
4

#### 2.3.8 Encapsulation Efficiency

The amount of extract encapsulated in bilayer wound dressing was determined by dissolving a certain amount of nanofiber layer in 1× PBS solution. First, chitosan nanofibers were dissolved in an ultrasonic bath for 30 min to destroy the polymer structure. The amount of *C. quadrangularis* released from the nanofibers was measured with a UV spectrophotometer (Varioskan) at 230 nm wavelength. The encapsulation efficiency (EE %) was calculated with eq 5.
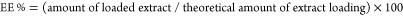
5

### 2.4 *In Vitro* Extract Release Profile

Bilayer wound dressings were placed on a 24-well plate and incubated in 1 mL of 1× PBS solution. Samples were incubated at 37 °C in an orbital shaker at 55 rpm (Thermoshake, Gerhardt). The release rate of the samples collected at certain time intervals was determined with the absorbance data obtained using a UV spectrophotometer at 230 nm. At every detection time, fresh 1× PBS solution was added to the collected material to maintain the consistent volume of the incubation medium. In this study, first-order, Higuchi, and Korsmeyer–Peppas release models were used to determine the kinetics and the dominant mechanism of CQ release from the bilayer wound dressing.

### 2.5 Antimicrobial Activity

CQ release media were collected from the *in vitro* release study at predetermined time periods of 1, 6, and 24 h and used for antimicrobial activity test by the disc diffusion method. CQ release media were tested on Gram-positive *Staphylococcus epidermidis* (RSKK 1009 strain) and Gram-negative *Escherichia coli* (ATCC 25922). Cultures were activated in nutrient broth for 24 h at 37 °C before use. The bacterial concentration was set at 0.5 McFarland. Then, the bacterial solution was spread on agar for cultivation. Blank discs (Oxoid) were placed on the cultivated Petri dish. Then, 10 μL of extract release media collected at specific times (1 and 6 h) was dropped on blank discs. Amoxicillin (antibiotic) discs were tested as positive control groups. Petri dishes were incubated at 37 °C for 24 h. Then, clear inhibition zones were measured and recorded as the average of four replicates.

### 2.6 *In Vitro* Cell Culture

#### 2.6.1 *In Vitro* Wound Healing with Scratch Assay

*In vitro* scratch assay was carried out with the NIH/3T3 fibroblast cell line. Cells were cultured on both pre-coated and noncoated wells to observe the wound healing effect of *C. quadrangularis* extract on both cell migration and proliferation, respectively. Scratch assay was performed on 96-well plates, and the wound area was mechanically created. Cells were cultivated at 37 °C, and DMEM culture medium (2 mM l-glutamine, 10% fetal bovine serum, 100 μg/mL streptomycin, and 100 U/mL penicillin) was used in an atmosphere of 5% CO_2_. *C. quadrangularis* extract with different ratios (2.5:1, 5:1, and 7.5:1) was dissolved in DMEM as extraction medium. FBS (1%) was used to minimize the cell proliferation. Well plates were coated with the ECM substrate poly-l-lysine to obtain cell–ECM interaction and prevent cell motility for migration assay. Fibroblast cells were seeded on each well of 96-well plates with a density of 10^5^ cell/mL and incubated overnight to obtain a confluent cell monolayer. After incubation, a 200 μL pipette tip was used to scratch a linear wound on the cell monolayer. Then, PBS solution was used to wash the cellular debris. Scratched cell monolayers were incubated with extraction media. The control group (untreated) was incubated with only DMEM (1% FBS). Each well was observed with a microscope (Olympus CX 31) at different incubation periods (0, 24, and 48 h). The scratch area was observed using an Olympus DP 25 with 4× magnification. Wound closure was calculated by analyzing the images with Olympus DP2 BSW Software at different incubation times using eq 6, where *W*_0_ is the initial wound width and *W*_t_ is the wound width at the incubation period.
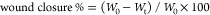
6

#### 2.6.2 *In Vitro* Cytotoxicity Determination

The cytotoxicity of single-layer and bilayer composite sponges was determined according to ISO 10993-5 using WST-1 assay. NIH/3T3 fibroblast viability was measured using an indirect extraction method. Extract-loaded nanofibers (upper single layer) and extract-loaded coated CHI-POSS sponges (bilayer) were extracted in DMEM at 24 h at 37 °C. NIH/3T3 cells on a 96-well plate with a density of 10^5^ cell/mL were incubated with 100 μL of extraction media for 72 h. Cell viability % was determined during 72 h spectrophotometrically at 440 nm and calculated according to eq 7 (ABS: average absorbance value; NC: negative control) by normalizing the absorbance data with a negative control that includes fresh medium.
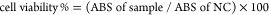
7

#### 2.6.3 Cell Attachment and Spreading

NIH/3T3 and HS2 cells were seeded on porous and fiber layers and observed at 7 days for cell attachment and spreading. Cells were fixed with 3.7% paraformaldehyde (v/v) in PBS solution for 20 min at room temperature. Then, samples were washed with 1× PBS solution. Cell permeability was obtained with 0.1% Triton X-100. Cell nuclei and cytoskeleton were stained with DAPI and Alexa Fluor 488 and visualized with fluorescence microscopy (ZEISS Observer Z1). For SEM analysis, fixed samples were dehydrated with ethanol series (20, 50, 75, and 100%) and dried at 40 °C before imaging.

#### 2.6.4 Cell Proliferation

NIH/3T3 and HS2 cell lines were used to mimic the skin tissue, and each cell line was seeded on porous and fiber layers of scaffolds. DMEM culture media (10% FBS, 1% l-glutamine, and 1% penicillin–streptomycin) were used for cell cultivation. Scaffolds were neutralized in 1 M NaOH for 30 min and incubated in 70% (v/v) ethanol solution overnight for sterilization. Scaffolds were washed thrice with 1× PBS solution and conditioned with cell culture medium for 2 h before cultivation. NIH/3T3 and HS2 cells were seeded on each layer of scaffolds at a density of 2 × 10^6^ cell/mL. Then, scaffolds were incubated 4 h at 37 °C for cell attachment. The culture medium was changed thrice a week. The WST-1 cell viability kit was used to detect cell proliferation. Absorbance data were measured with a plate reader (Varioskan Flash, Thermo Fisher Scientific) at 440 nm wavelengths.

#### 2.6.5 Determination of Hydroxyproline (HP)

Total hydroxyproline content in collagen matrix formation on bilayer sponges was determined on the 14th day with the colorimetric HP assay kit (Elabscience) according to the manufacturer’s protocol (*n* = 3). Colorimetric measurement was obtained spectrophotometrically at 550 nm.

#### 2.6.6 Glycosaminoglycan (GAG) Content

GAG production of NIH/3T3 cells on scaffolds was determined with the proteoglycan detection kit (Amsbio, AMS Biotechnology) at 7 and 14 days of incubation (*n* = 3). The papain extraction method was used to extract GAG content from samples. Scaffolds were incubated with papain solution (papain, dithiothreitol (DTT), and EDTA) for 6 h at 60 °C. After extraction, DMMB (dimethyl methylene blue) solution was added, and GAG content was measured spectrophotometrically at 530 nm.

#### 2.6.7 Determination of Collagen (Type I) Secretion

Col1A1 secretion of NIH/3T3 cells on bilayer wound dressings was measured using the Human COMP Sandwich ELISA assay (Elabscience) according to the manufacturer’s protocol (*n* = 3). The culture media of scaffold groups were extracted for 7 and 14 days of incubation.

### 2.7 Statistical Analysis

Experiments were performed in triplicate. The experimental data of characterization tests are presented as the mean ± standard deviation (SD). Statistical analyses of *in vitro* studies were presented as the standard error of mean. The statistical differences between experiment groups were analyzed using a two-way analysis of variance (ANOVA) with Tukey’s multiple comparison test.

## 3 Results and Discussion

### 3.1 Morphology and Structure of Composite Sponges (Bottom Layer)

**Figure 1:**
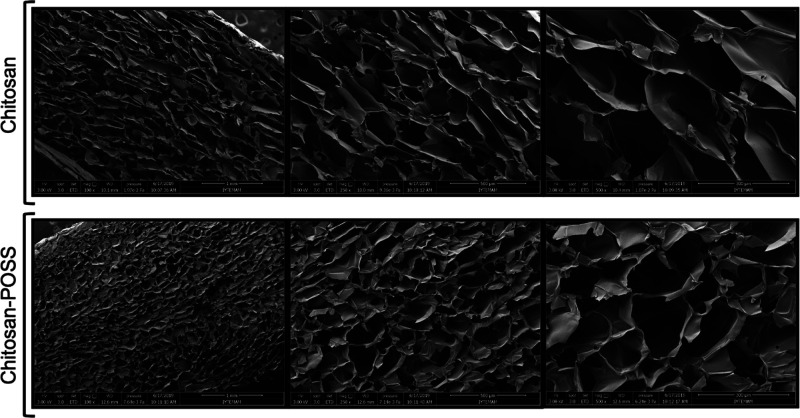
SEM images of chitosan and chitosan-POSS composite sponges with 100×, 250×, and 500× magnifications.

The pore structure, dimensions, and morphology of the sponges were observed with SEM analysis. Figure 1 shows SEM images indicating the microstructure of CHI and CHI-POSS composite sponges. Pore sizes of the CHI and CHI-POSS sponges were measured using ImageJ Software and found to be 163 ± 7 and 137 ± 8 μm, respectively. The average pore size of CHI-POSS sponges decreased when POSS nanoparticles were incorporated into the chitosan matrix. In addition, CHI-POSS composite sponges showed a more homogeneous pore structure compared to chitosan sponges. In the literature, Park et al. fabricated single-layer chitosan-Si membranes using the sol–gel method and freeze-casting process. Chitosan-Si membranes were fabricated with an average pore size of 200–250 μm.^12^ In addition, Tamburaci and Tihminlioglu fabricated chitosan/POSS nanocomposite sponges for bone tissue regeneration. The average pore size of chitosan-POSS scaffolds was observed to be in a range of 150–190 μm. However, at high POSS concentrations (20–40%), the average pore size decreased, the morphology of scaffolds was altered, and pore wall surfaces were enlarged.^26^

### 3.2 Morphology and Structure of Nanofibers (Upper Layer)

**Figure 2:**
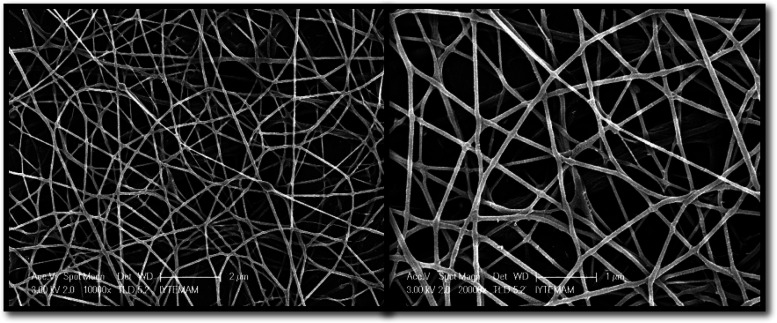
SEM images of the chitosan fiber layer.

**Figure 3:**
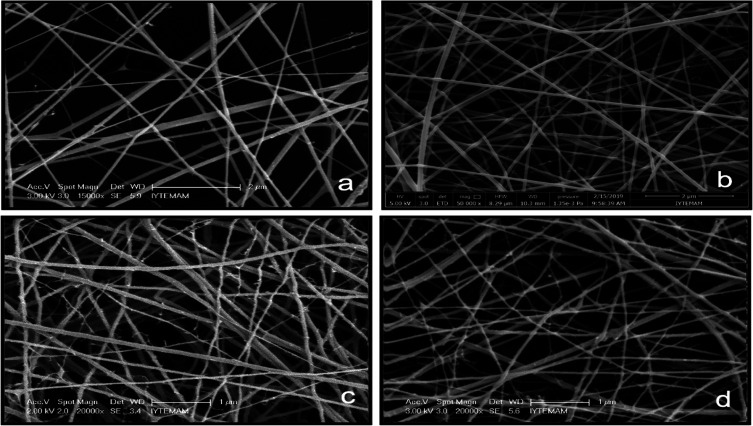
SEM images of nanofibers coated on chitosan-POSS sponge with different genipin concentrations (wt %): (a) 0.5, (b) 1, (c) 2, and (d) 3.

According to the SEM images, it was observed that fiber formation was obtained with uniform morphology (Figure 2). Genipin was used as a crosslinker at different concentrations (0.5, 1, 2, and 3% (w/w CHI)) to enhance the stability of chitosan fibers. Figure 3 shows the morphology of chitosan nanofibers at different genipin concentrations. The morphologies of the fiber groups containing 0.5 and 1% genipin (w/w CHI) were shown to be smooth, while the structure of the nanofibers grew thinner as the genipin concentration rose. The fiber diameters for the groups with genipin concentrations of 0.5, 1, 2, and 3% (w/w CHI), respectively, were determined to be 89.95, 77.94, 55.13, and 50.75 nm.

**Figure 4:**
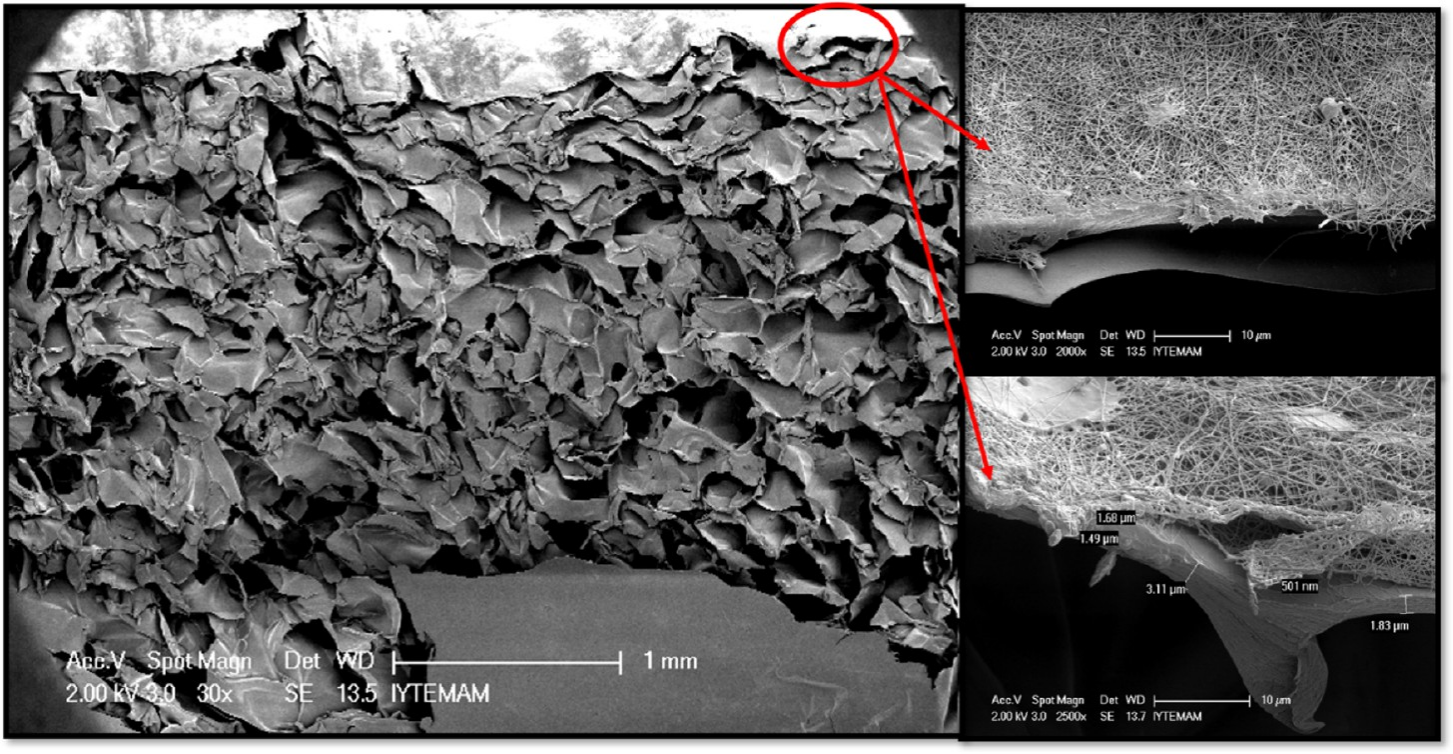
SEM image of a cross section of CQ-loaded chitosan nanofiber-coated bilayer sponges.

Nanofibers are coated on the sponge by using 1 mL volume of extract-loaded solution. As seen in Figure 4, nanofibers are coated uniformly and covered on the sponge. The coating thickness of the nanofiber layer was measured as 1.5 ± 0.2 μm. The fiber thickness coated on the sponge layer was found suitable to mimic the epidermis layer. On the other hand, the thickness of sponges was measured as 3.02 mm by ImageJ software. Since the thickness of dermis is in the range of 1–3 mm according to the body region,^22,26^ thus, the thickness of the fabricated sponge layer was found appropriate to represent the dermis layer of skin tissue.

**Figure 5:**
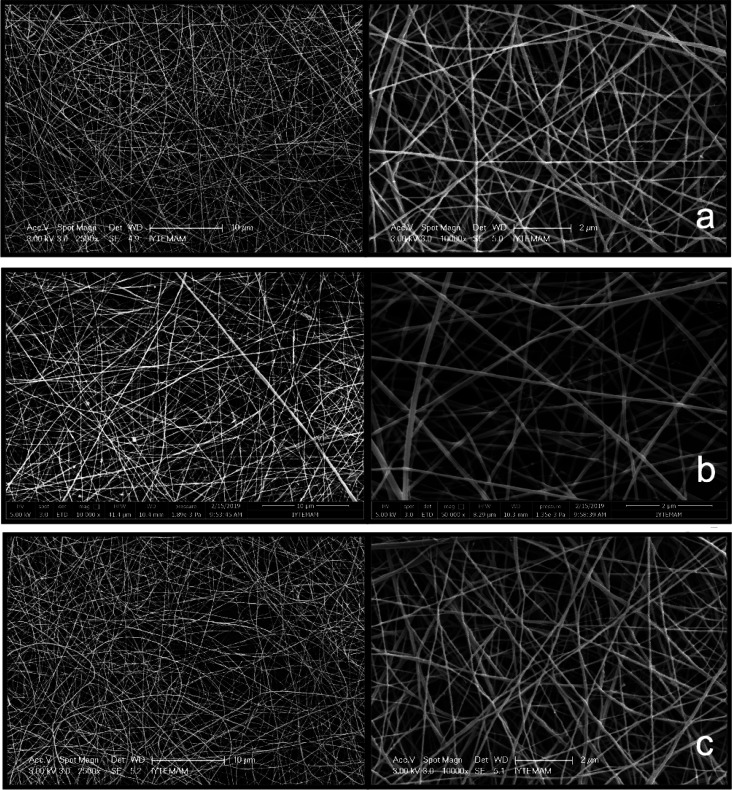
SEM image of extract-loaded chitosan nanofibers coated on the sponge at different P:E ratios: (a) 2.5:1, (b) 5:1, and (c) 7.5:1.

Nanofibers were fabricated at three different polymer:extract ratios, P:E’s (P:E of 2.5:1, 5:1, and 7.5:1). Different extract ratios did not significantly affect the structure and morphology of nanofibers (Figure 5). Fiber diameters were found to be 97.4 ± 4, 77.9 ± 4, and 83.2 ± 3 nm in groups with P:E values of 2.5, 5:1, and 7.5, respectively. Similarly, in a study, the average diameter of chitosan fibers produced by the electrospinning method was found to be 100–130 nm.^27^ In another study, Li and Hsieh have produced chitosan-based nanofibers that are in the diameter size range of 20–100 nm.^28^ Type I collagen fibril diameters vary between 50 and 500 nm in native skin tissue.^29^ Therefore, in this study, the average nanofiber diameters were found to be in the appropriate range to mimic the structure of the ECM.

### 3.3 *In Vitro* Release Profile

**Figure 6:**
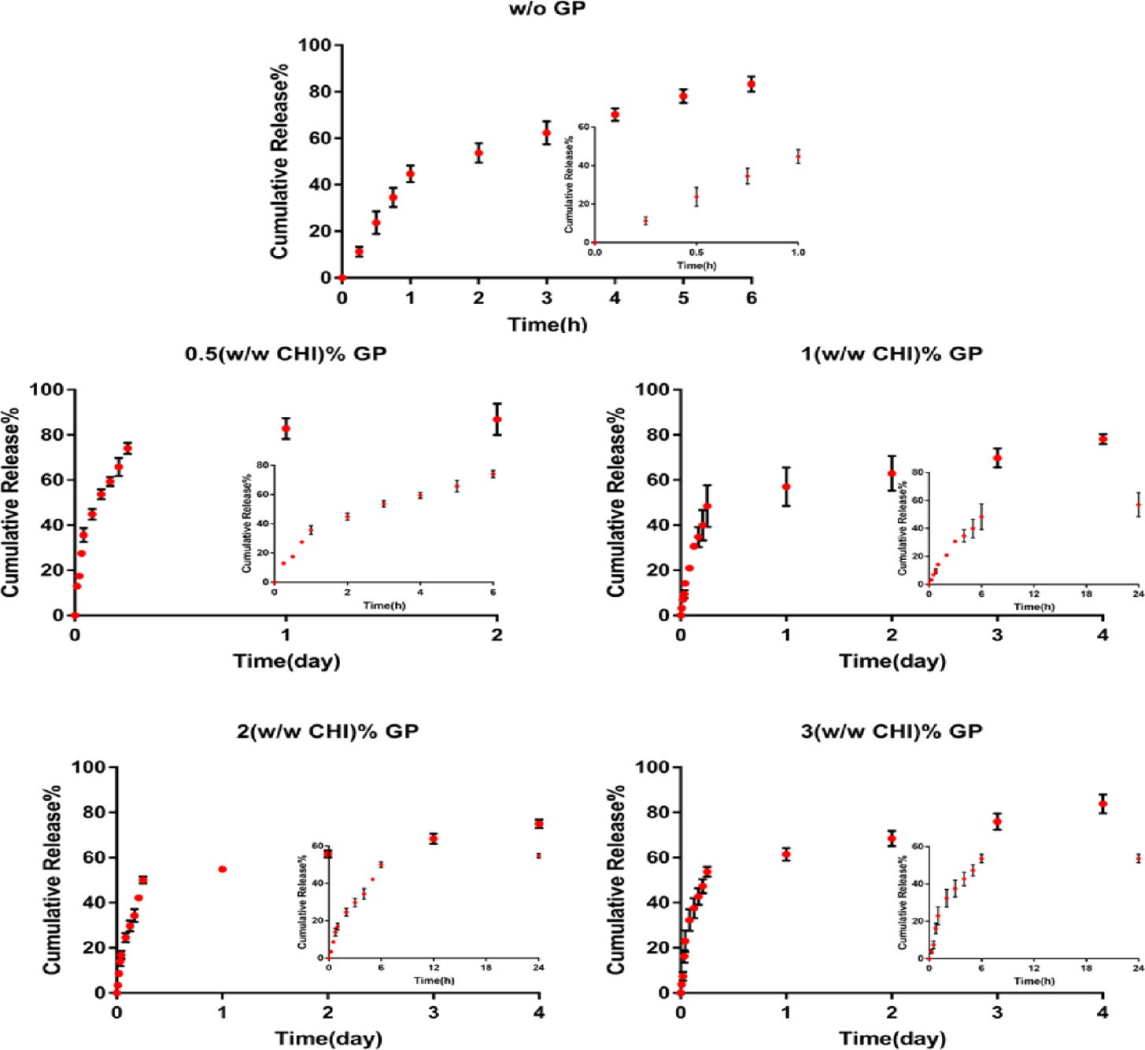
Cumulative release of the CQ extract from nanofiber-coated sponges without genipin and with different genipin concentrations.

Before *in vitro* release experiments, the encapsulation efficiency of each nanofiber group was determined before coating on the sponge surface. The effect of different P:E ratios on encapsulation efficiency in nanofiber production was investigated. With increasing P:E ratio, the encapsulation efficiencies of CQ-loaded nanofibers were 60.00 ± 1.78, 70.17 ± 2.12, and 76.86 ± 1.98%. After the encapsulation efficiency was calculated, the *in vitro* release of the CQ-loaded nanofiber-coated sponges was examined. The initial burst release of CQ extract from the uncrosslinked fiber layer was 81% of the extract at 6 h. The crosslinking effect on CQ extract release from the nanofiber layer was investigated at four different genipin ratios (0.5, 1, 2, and 3% (w/w CHI)) and a constant P:E ratio (5:1) (Figure 6). As the effect of the crosslinking ratio was evaluated, it was found that crosslinking slowed down the release rate; however, no significant differences between 1% genipin-crosslinked nanofibers and higher crosslinking ones (2 and 3% crosslinked nanofibers) were obtained. Therefore, the 1% (w/w CHI) genipin ratio was found to be suitable for chitosan nanofiber fabrication.

**Figure 7:**
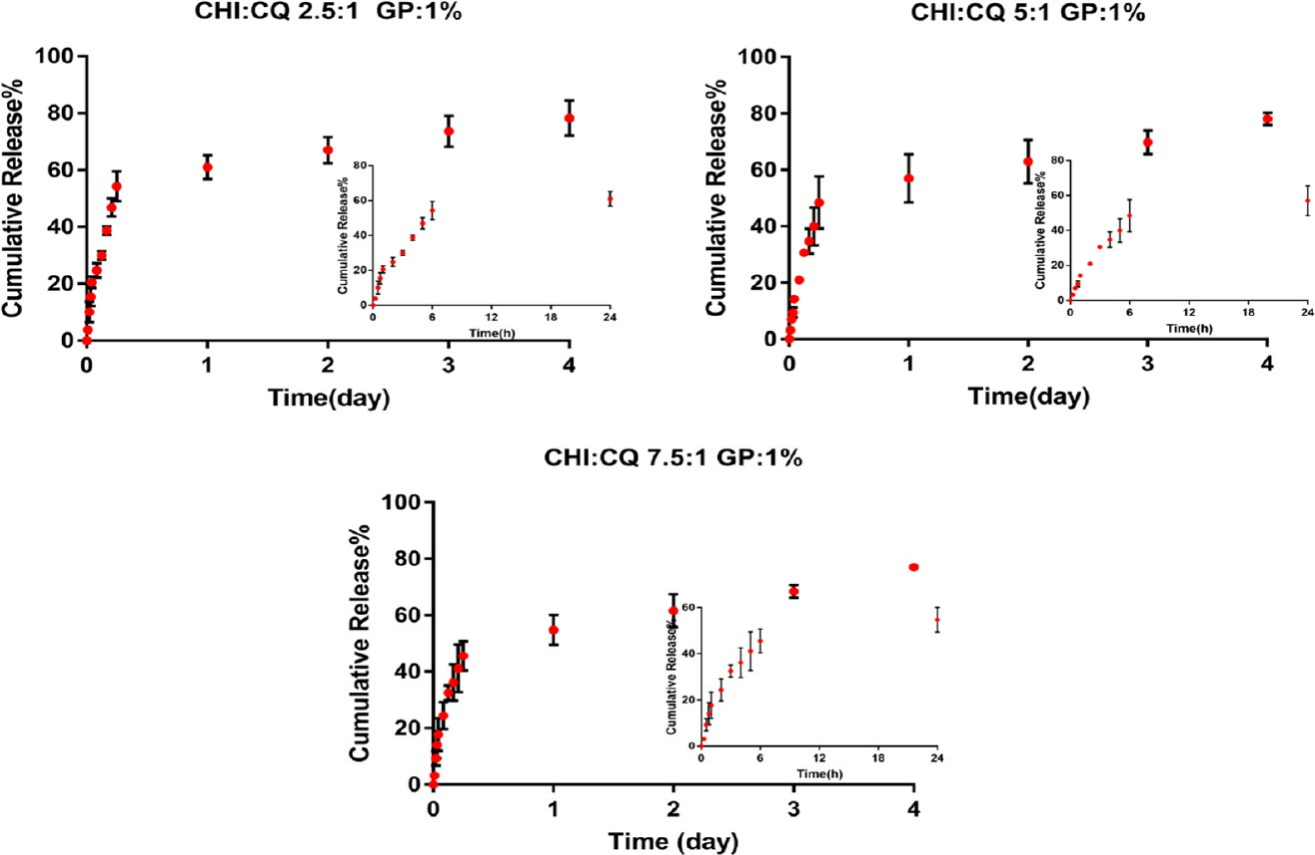
Cumulative release of extract from nanofiber-coated sponge for three different extract ratios (P:E ratio: 2.5:1, 5:1, and 7.5:1).

The effect of polymer:extract ratio (P:E) on the release rate was also investigated. Three different extract ratios (2.5:1, 5:1, and 7.5:1) were used at constant genipin concentration (1% (w/w CHI). Bilayer sponges coated with CQ extract-loaded fibers at different concentrations showed similar release behavior (Figure 7). Approximately 50% of the extract was released from bilayer wound dressings in 6 h and reached 78% cumulative release on the 4th day of incubation for all extract-loaded fiber-coated wound dressings. This may arise from the retarding effect of the porous CHI-POSS sublayer. Similarly, Kiadeh et al. fabricated bilayer wound dressing composed of asymmetrical PCL membranes coated with a lidocaine-loaded chitosan-silica matrix. *In vitro* release results indicated that the final drug release from the bilayer wound dressing was found to be 72.5 ± 0.8% on the 4th day and the porous chitosan-silica matrix coating decreased the initial burst drug release in the bilayer PCL-PEG membrane.^30^ In this study, CQ was used as a bioactive herbal extract and, according to our knowledge, there is no study in the literature related to the encapsulation of CQ extract into the polymer matrix as a sustained-release system. In addition, there is limited literature knowledge about the use of CQ extract for wound healing applications. In the literature, the release of *Garcinia mangostana* extract-loaded chitosan-ethylenediaminetetraacetic acid/polyvinyl alcohol (CS-EDTA/PVA) mats was analyzed. Results indicated that 80% of extract was released in 60 min. At the end of 8 h, it reached 90% cumulative release.^31^ In another study, curcumin-loaded chitosan/gelatin sponges showed 30% burst release in the first 6 h. Extract-loaded sponges reached 90% cumulative release at the end of 4 days.^32^ In the literature, Andra and co-workers fabricated a *C. quadrangularis* (CQ)- and *Galinsoga parviflora* Cav (GP)-loaded PVA electrospun matrix. *In vitro* release studies of CQ extract from the PVA mat were carried out. Results indicated that 60% of the CQ extract was released in the first 8 h.^33^

Mathematical relations regarding the extract release were utilized to predict the release rate at the designated time. First-order, Higuchi, and Korsmeyer–Peppas models were applied to investigate release kinetics and the mechanism of CQ release from the bilayer wound dressing. First-order and Higuchi models were separately applied for two different regions of release data. The first zone, known as the burst release, is composed of the first 6 h of the release data, while the second region, known as the sustained release, is made up of the release data between the first and fourth days. The Korsmeyer–Peppas model is a semi-experimental model that assumes that diffusion is the main mechanism that controls the release profile from polymeric systems. In general, this model is applied by placing the first 60% of cumulative release in the model. It was found that first-order, Higuchi, and Korsmeyer–Peppas models fit the extract release data according to their high *R*^2^ values (Table 1). When literature studies were examined, the Korsmeyer–Peppas model best described the drug or extract release from the chitosan fiber-based polymer system.^31,32,34^

**Table 1:** Release Kinetic Coefficients for Chitosan-Based Bilayer Wound Dressing

		CHI:CQ 2.5:1		CHI:CQ 5:1		CHI:CQ 7.5:1	
model		burst release (6 h)	sustained release (1–4 days)	burst release (6 h)	sustained release (1–4 days)	burst release (6 h)	sustained release (1–4 days)
first-order	*R*^2^	0.9793	0.9948	0.9914	0.9694	0.9643	0.9868
Higuchi	*R*^2^	0.9549	0.9935	0.9573	0.9703	0.9901	0.9889

model		first 60% extract release		
Korsmeyer–Peppas	*R*^2^	0.9563	0.9792	0.9291

### 3.4 Antimicrobial Activity

**Figure 8:**
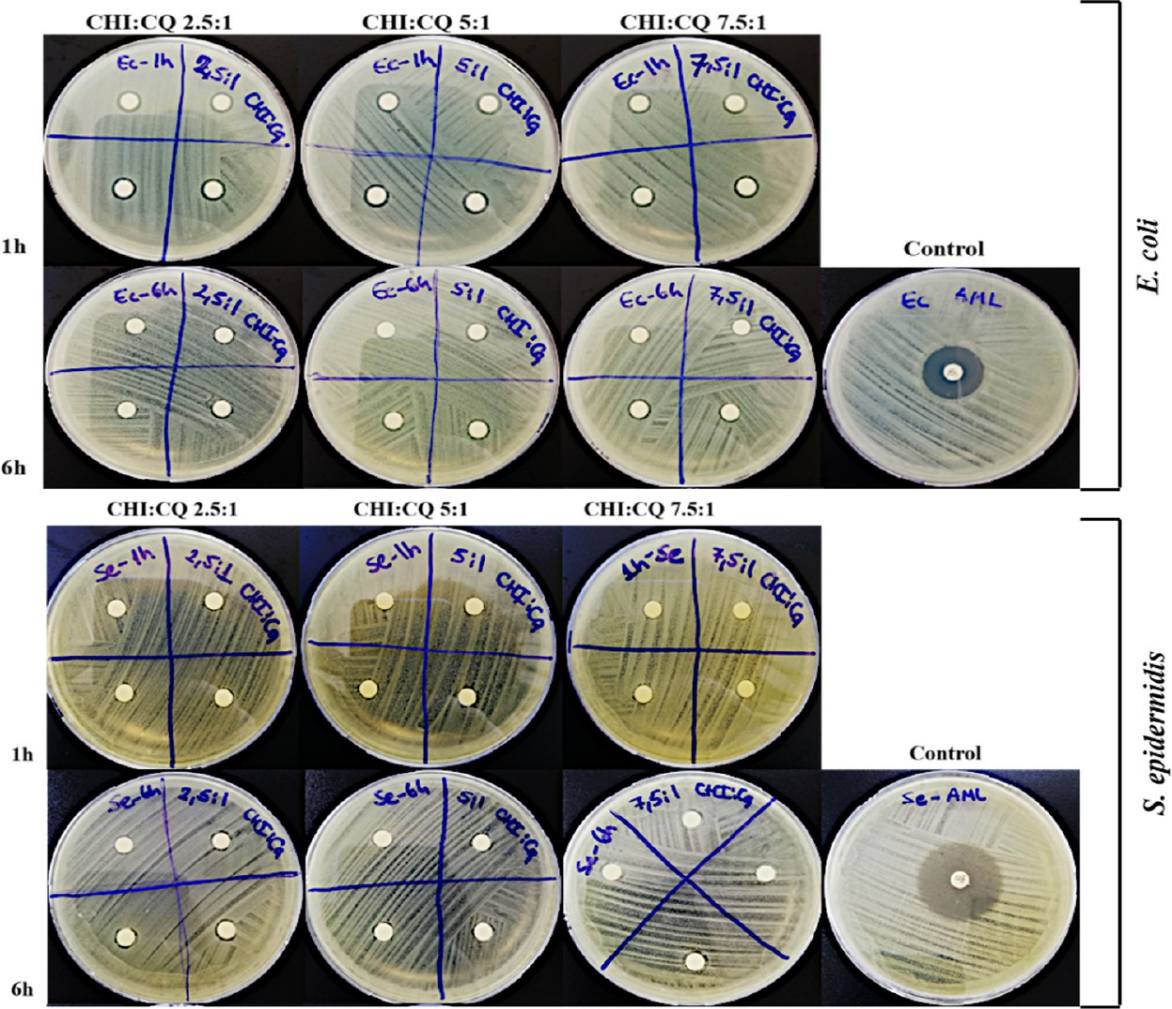
Effect of release media against *E. coli* and *S. epidermidis* at incubation times of 1 and 6 h.

One of the most effective bacteria in wound infections is known as *S. epidermidis*. This bacterium can be found in the peripheral skin and is frequently seen in wound infections. Another bacterium that is effective in wounds such as traumatic wounds and burn wounds is *E. coli*. These bacteria can cause infection in the wound area due to environmental causes.^35,36^ The use of antibiotics or bioactive extracts not only promotes wound healing but also protects the wound from bacterial infections. Several research investigated the possible mechanisms of plant constituents, namely, alkaloids, phenols, flavonoids, triterpenoids, and others, that induce antimicrobial activity.^37^ Thus, CQ was used as herbal extract to support wound healing and to prevent bacterial infections in the wound area. The antimicrobial activity of *in vitro* release media obtained from bilayer dressings was tested on Gram-negative bacteria *E. coli* and Gram-positive bacteria *S. epidermidis*. Release media of bilayer dressing showed antimicrobial zones on both two pathogens at 1 and 6 h (Figure 8). Release media of bilayer wound dressings and three different ratios of ethanolic CQ extract showed effective antimicrobial activity in the first hours for both pathogens due to the burst release of the extract. In addition, it was found that zone diameters obtained against *E. coli* and *S. epidermidis* increased with increasing CQ extract ratio in bilayer wound dressings (Table 2). It was also observed that the release media from bilayer dressings formed a larger zone diameter against *E. coli* compared to *S. epidermidis.* In the literature, it was observed that CQ extract showed antimicrobial activity against both Gram-negative and Gram-positive bacteria.^38,39^ A recent study investigated the bioactive components of stems of CQ and the antimicrobial activity of methanol and ethanol extracts of CQ. Phytochemical analysis of stems of CQ indicated that CQ is composed of saponin, tannin, phenol, flavonoid, terpenoid, and alkaloids in aqueous, ethanol, and methanol extracts. At the tested doses of 100, 200, 300, and 400 g, the results also showed that ethanol and methanol extracts of CQ demonstrated highly effective antimicrobial activity against avian microorganisms, including *E. coli*, *Klebsiella*, *Salmonella*, *Pasteurella*, *Staphylococcus*, and *Aspergillus* species.^40^ Andra et al. investigated the antimicrobial effect of CQ extract loading in PVA electrospun nanofiber mats and observed that CQ showed antibacterial activity against *E. coli* and *Staphylococcus aureus*. In addition, disc diffusion results indicated that the antibacterial activity increased as the concentration of the CQ extract increased.^33^

**Table 2:** Effect of *In Vitro* Release Media (1, 6, and 24 h) against *E. coli* and *S. epidermidis*

	inhibition zone diameter (mm)	
group	1 h	6 h
*E. coli*		
positive control (amoxicillin)	8	8
CHI:CQ 2.5:1	1.62 ± 0.08	1.4 ± 0.1
CHI:CQ 5:1	1.00	0.96 ± 0.03
CHI:CQ 7.5:1	1.00	0.83 ± 0.08
*S. epidermis*		
positive control (amoxicillin)	11	11
CHI:CQ 2.5:1	1 ± 0.1	0.76 ± 0.12
CHI:CQ 5:1	0.82 ± 0.09	0.7 ± 0.13
CHI:CQ 7.5:1	0.88 ± 0.07	0.65 ± 0.92

### 3.5 Fourier Transform Infrared Spectroscopy (FT-IR) Analysis

**Figure 9:**
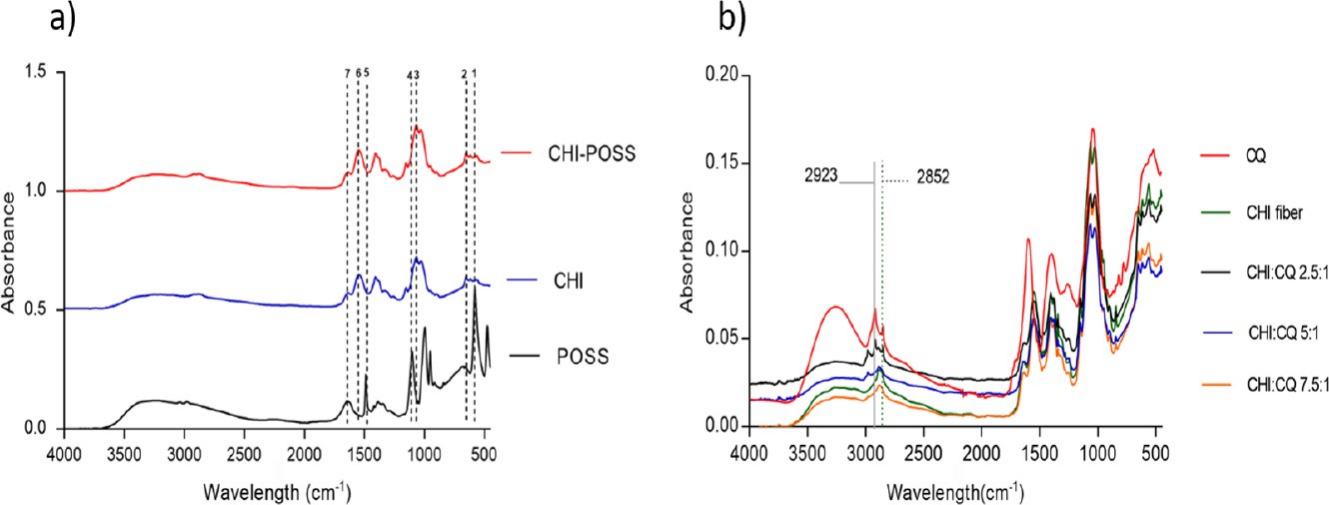
(a) Characteristic bands of CHI and POSS and (b) FT-IR spectra of CHI fiber-coated bilayer sponges with different P:E ratios.

FT-IR analysis was performed to indicate the characteristic peaks of chitosan, CQ, and POSS as well as the chemical interaction between chitosan-POSS and chitosan-CQ extract. Chitosan-POSS sponges showed characteristic peaks of both chitosan and POSS structures. As seen in Figure 9a, the main characteristic peaks of chitosan are C=O stretching (amide I) at 1651 cm^–1^ wavelength, N–H bending (amide II) at 1551 cm^–1^ wavelength, and C–N stretching and N–H bending of amide linkages at 1380 cm^–1^.^40^ When the characteristic peaks of POSS are examined, the Si–O–Si stress bands are seen at wavelengths of 580, 1006, and 1095 cm^–1^. The vibrations of the reactive groups of the POSS nanocage (tetramethyl ammonium) appeared at 1497 and 1651 cm^–1^ in Figure 9a.^41^ CHI-POSS composite sponges showed a slight shift in Si–O–Si peaks at 555–650 cm^–1^ and 1070 cm^–1^ with stretching and bending vibrations, respectively. The main characteristics of chitosan and POSS are depicted in Table 3. The FT-IR spectrum of *C. quadrangularis* powder consists of characteristic bands that appeared at 2922 and 2850 cm^–1^ (C–H stretching), 3220 and 3290 cm^–1^ (OH stretching), between 1600 cm^–1^ (C=O aromatic stretching) and 1396 cm^–1^ (C–O stretching of phenol and ester), and 1036 cm^–1^ (the alkoxy C–O band).^39,40^Figure 9b shows the FT-IR spectra of CQ extract-loaded bilayer sponges with different polymer/extract ratios as well as chitosan fiber-coated bilayer sponges as a control group. The main peaks of chitosan appeared for all samples; however, the characteristic peaks of CQ (at 2923 and 2852 cm^–1^) were only observed for extract-loaded coating with high concentration (P:E ratio of 2.5).

**Table 3:** Characteristic Peaks of Chitosan and POSS

no.	wavenumber (cm^–1^)	band	formulation
1	580	Si–O–Si stretching	POSS
2	650	Si–O–Si stretching	POSS
3	1070	Si–O–Si bending	POSS
4	1095	Si–O–Si stretching	POSS
5	1497	tetramethylammonium	POSS
6	1551	N–H bending (amide II)	CHI
7	1651	C=O stretching (amide I)	CHI
8	1651	tetramethylammonium	POSS

### 3.6 Swelling Study

**Figure 10:**
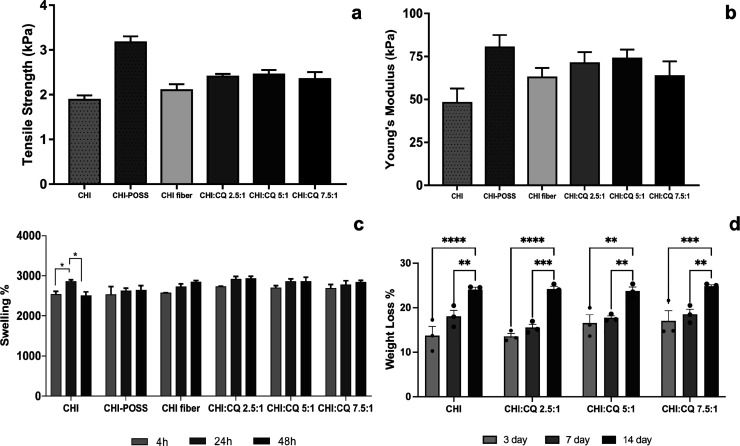
(a) Young’s modulus of single-layer and bilayer wound dressings, (b) tensile strength of single-layer and bilayer wound dressings, (c) swelling percentages of single-layer and bilayer wound dressings for 4, 24, and 48 h, and (d) weight loss % of bilayer wound dressings for 3, 7, and 14 days of incubation.

Swelling tests were performed to specify the water uptake capacity of single-layer and bilayer wound dressings at the wound site. Swelling tests were performed with respect to weight changes of samples at 4, 24, and 48 h in 1× PBS solution. Figure 10c shows the swelling percentages of single-layer and bilayer wound dressings. According to the swelling test results, the water absorption capacity of chitosan increased at 24 h and decreased at 48 h due to the low stability of chitosan. A statistical analysis revealed a significant difference in the swelling percentage of the chitosan control group (CHI) at 4 and 24 h as well as at 24 and 48 h. But adding POSS nanoparticles to the chitosan matrix increased the swelling percentage during all incubation durations. Bilayer dressings showed the same swelling pattern at 4, 24, and 48 h. Since the nanofiber layer is much thinner than the sponge layer, bilayer wound dressings do not result in an increase in swelling percentage. Similarly, Park and co-workers fabricated Si-loaded chitosan membranes for skin tissue regeneration and investigated the surface hydrophilicity of CTS-Si membranes with an immersion method. Results indicated that CTS-Si membranes can enhance the wound exudate sorption due to the higher water sorption capacity of Si compared to a neat CTS membrane.^12^

### 3.7 Open Porosity Determination

The open porosity of the single-layer and bilayer wound dressings was measured using the liquid displacement method. Table 4 displays the single-layer and bilayer wound dressings’ open porosity percentages. There is little difference between the open porosity of single-layer sponges and that of bilayer sponges. Because POSS nanoparticle incorporation increased the pore wall surface and thickened the sponge walls, the open porosity of the chitosan composite slightly decreased. Due to the thin fiber layer coating, it was observed that the open porosity of the double-layer sponges was comparable to that of the single-layer chitosan-POSS composite sponges.

**Table 4:** Open Porosity Percentages of Single-Layer and Bilayer Sponges

group	open porosity (%)
CHI sponge	81.23 ± 1.38
CHI-POSS sponge	77.51 ± 3.43
CHI fiber	78.33 ± 2.00
CHI:CQ 2.5:1 fiber	78.93 ± 2.67
CHI:CQ 5:1 fiber	79.36 ± 3.96
CHI:CQ 7.5:1 fiber	77.56 ± 1.94

### 3.8 Water Vapor Transmission Rate

Water vapor permeability is a significant property in wound healing affecting the moisture in the wound area. The dressing should absorb excess fluid from the wound site. The wound should be kept at the appropriate humidity. Normal human skin has a water vapor permeability of 204 g/m^2^/day. The ideal wound dressing is considered to have a water vapor permeability value of 279–5138 g/m^2^, connected with the wound type.^42^ In this study, the water vapor permeability of chitosan, CHI-POSS sponge, and bilayer sponges was examined. Table 5 gives experimental permeability values of single-layer chitosan, chitosan-POSS sponge, and the bilayer sponge loaded with three different CQ extract ratios. The water vapor permeability of single-layer and bilayer sponges was determined to be within the ideal range for wound dressings.

**Table 5:** WVP and WVTR Values of Single-Layer and Bilayer Sponges

group	WVP × 10^5^ (mol min^–1^ cm^–1^ kPa^–1^)	WVTR (g m^–2^ day^–1^)
CHI	2.95 ± 0.15	4251.97 ± 150
CHI-POSS	3.15 ± 0.54	4609.06 ± 13
CHI:CQ 2.5:1	2.60 ± 0.5	4013.16 ± 36
CHI:CQ 5:1	2.60 ± 0.4	4021.29 ± 300
CHI:CQ 7.5:1	2.95 ± 0.05	4243.1 ± 330

The permeability of the sponges was reduced when coated with nanofibers. This is because the diffusion path is slightly higher than the monolayer sponges, so the permeability is reduced in the expected direction.^43^ At the same time, since the sponges have a porous structure, the nanofibers have caused these pores to be closed, and the closure of the pores has led to a decrease in permeability. In the study, the permeability of chitosan/gelatin hydrogels prepared for wound healing was examined and found to be 2228 ± 31.8 g/m^2^ day.^35^ It is very important for wound dressing to have appropriate water vapor permeability for wound healing. The bilayer dressings, fabricated in this study, are found to be in the ideal dressing range with higher permeability.

### 3.9 Mechanical Properties

Mechanical properties of single-layer and bilayer dressings were investigated with tensile strength and Young’s modulus. Figure 10a,b shows Young’s modulus and tensile strength results of single-layer and bilayer dressings. Young’s modulus values of single-layer and bilayer dressings are found to be in the range of 48.02 to 80.29 kPa. In the literature, Young’s modulus values of natural skin change between 5 and 140 kPa depending on the skin region of the body.^44^ Thus, bilayer membranes showed compatible mechanical properties with skin tissue. Generally, inorganic additives enhance the mechanical properties of polymers. Results also indicated that Young’s modulus and mechanical strength of the chitosan sponge matrix increased with POSS nanoparticle incorporation.

In the literature, CHI-POSS nanocomposite dense membranes were fabricated with the solvent casting technique. The chitosan membrane had a tensile strength of 34.37 MPa, whereas CHI-POSS nanocomposite membranes showed significantly higher tensile strength with increasing POSS ratio and attained a maximum value of 60.28 ± 7.07 MPa at 3% (w/w) POSS concentration. In our study, bilayer CHI-CQ nanofiber-coated CHI-POSS membranes possessed lower tensile strength and modulus values compared to dense membranes due their highly porous structure. In another study, Tamburaci and Tihminlioglu fabricated CHI-POSS nanocomposite sponge with low molecular weight CHI and different POSS ratios up to 40% and indicated that increasing POSS ratio enhanced the mechanical characteristics up to 20%. Compression moduli and mechanical strength of CHI-POSS scaffolds were found to be in the range of 13.5–20 kPa.^45^ In this study, higher values of Young’s modulus for medium molecular weight CHI and CHI-POSS sponges were found to be 48 and 80 kPa, respectively. Higher modulus data arise from the difference in molecular weight of chitosan. This change also led to alterations in the 3D structure, pore morphology, and interconnections.

### 3.10 Enzymatic Degradation

Chitosan and CQ-loaded chitosan nanofiber-coated CHI/POSS sponges were investigated as bilayer samples for enzymatic degradation study. Results indicated that all bilayer groups showed a similar degradation trend for 14 days of incubation (Figure 10d). At the end of the incubation period, 24% of the initial weight of bilayer samples degraded. The change in weight loss % of each group with incubation time was found to be statistically significant. Tamburaci et al. also fabricated CQ-loaded chitosan/Na-carboxymethyl polyelectrolyte complex scaffolds with different CQ loading and investigated their enzymatic degradation. It was indicated that CQ extract loading increased weight loss for 14th and 21st days of degradation periods due to possible dissolution of the extract.^21^ However, in this study, CQ extract was loaded in the nanofiber layer and a controlled release profile was obtained. Thus, CQ extract loading did not have a significant effect on the degradation profile.

### 3.11 *In Vitro* Cell Culture Studies

*In vitro* cell culture studies were performed with two different cell lines to mimic epidermal and dermal layers of skin tissue. HS2 keratinocyte and NIH/3T3 fibroblast cell lines were cultivated on nanofiber and porous layers of bilayer wound dressings.

#### 3.11.1 Cytotoxicity Determination

*In vitro* cytotoxicity of wound dressings was determined with the indirect extraction method (ISO 10993-5) using WST-1 cell viability assay. Fibroblasts are known to be major cells that participate in the wound healing process, especially in the proliferation stage.^46^ According to the ISO 10993-5 standard, the cell viability of the biomaterial for nontoxic materials should be greater than 80% and could be approved as a good biocompatible material.^47,48^ In this study, NIH/3T3 fibroblast cells were used to investigate the *in vitro* cytotoxicity. Figure 18a shows the viability (%) of NIH/3T3 fibroblast cells incubated with the extraction media of single-layer and bilayer wound dressings for 24, 48, and 72 h incubation periods. Cell viability results indicated that not only single-layer but also bilayer wound dressings did not show any cytotoxic effects on fibroblast cells. In addition, it was observed that the cell viability of all groups incubated with extraction media was found at high levels. In a study, the cytotoxicity of CQ-loaded PVA nanofiber mats was tested with 3T3 fibroblast cells for 24 h of incubation. The cell viability of the extract-loaded PVA nanofiber mats was 98.52%.^33^

#### 3.11.2 *In Vitro* Wound Healing

**Figure 11:**
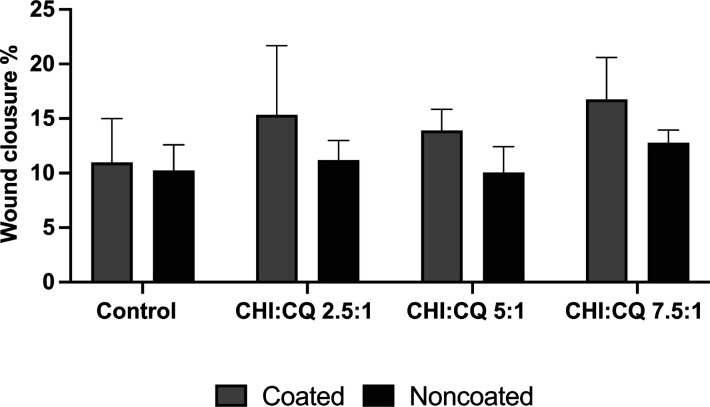
Wound closure % of fibroblast cell monolayers incubated with CQ extraction media.

**Figure 12:**
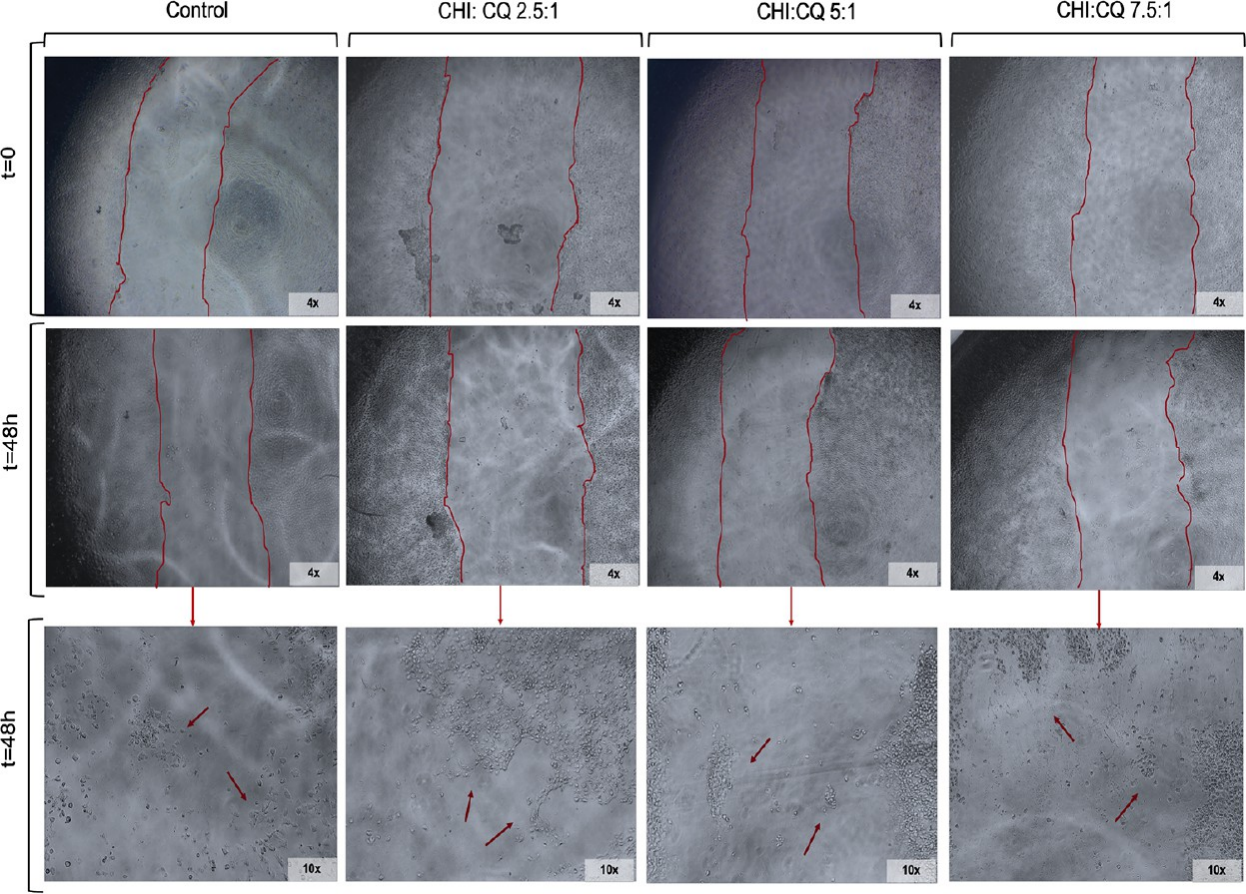
Microscopy images showing the wound boundaries of fibroblast monolayers in a 48 h period on poly-l-lysine-coated surfaces.

**Figure 13:**
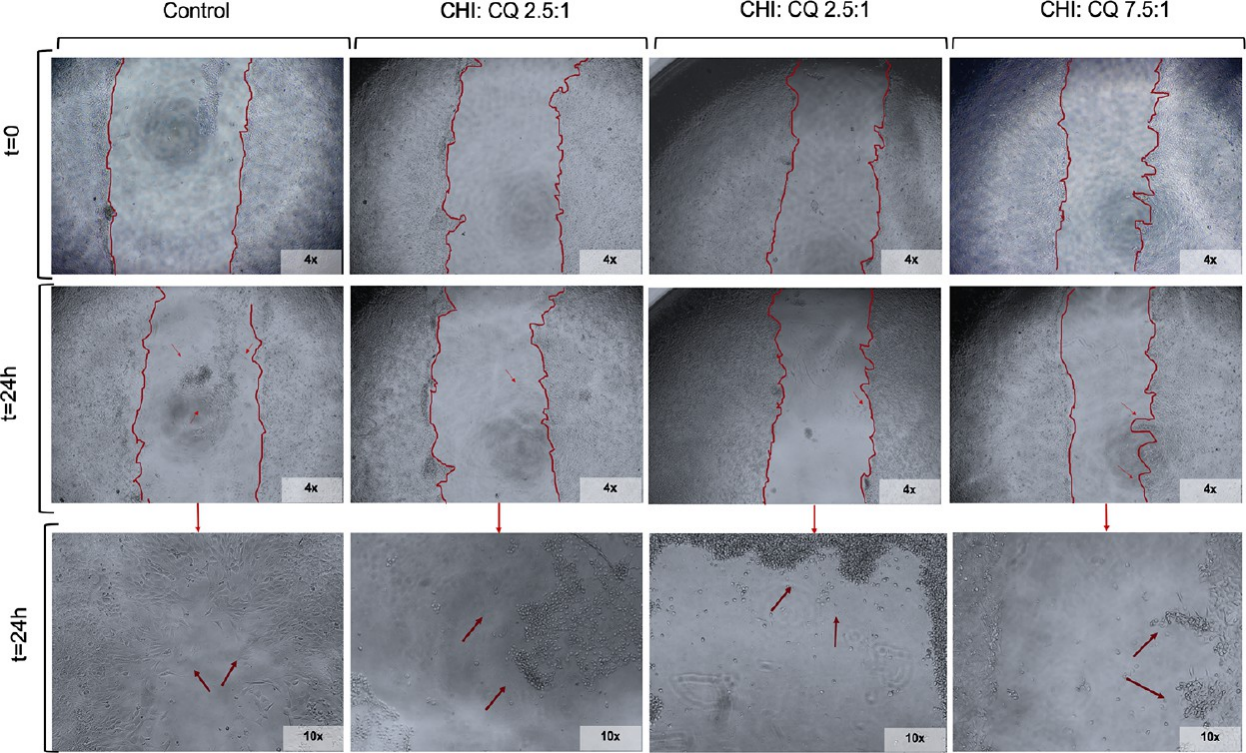
Microscopy images showing the wound boundaries of fibroblast monolayers in a 24 h period on noncoated surfaces.

The wound healing effect of *C. quadrangularis* extract was determined with *in vitro* scratch assay. The migration capacity of fibroblast cells was observed on poly-l-lysine-coated well plates, whereas the proliferation capacity of fibroblast cells at the wound area was observed on noncoated well plates. Wound closure % data obtained on fibroblast monolayers cultured with CQ extraction medium at various doses are shown in Figure 11. Microscopy images indicated that fibroblast cells that were located at the borders of the wound area migrated and proliferated through the gap with incubation of CQ extraction media (Figures 12 and 13). Results indicated that CQ extract media induced the cell migration at 48 h of incubation when compared to the control group. On noncoated surfaces, CQ extraction media positively affected cell proliferation at the boundaries of the wound area. However, increasing CQ concentration with a polymer (CHI)/CQ ratio from 7.5:1 to 2.5:1 did not cause a significant effect on cell proliferation compared to the control group.

#### 3.11.3 Cell Attachment and Spreading

**Figure 14:**
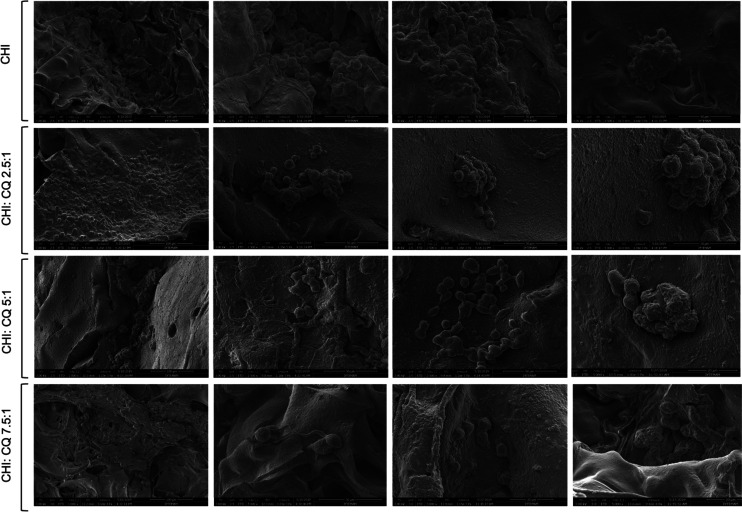
Scanning electron microscopy images of HS2 cells attached on CHI-CQ nanofiber layers on the 3rd day of incubation with 1000×, 2500×, and 5000× magnifications.

**Figure 15:**
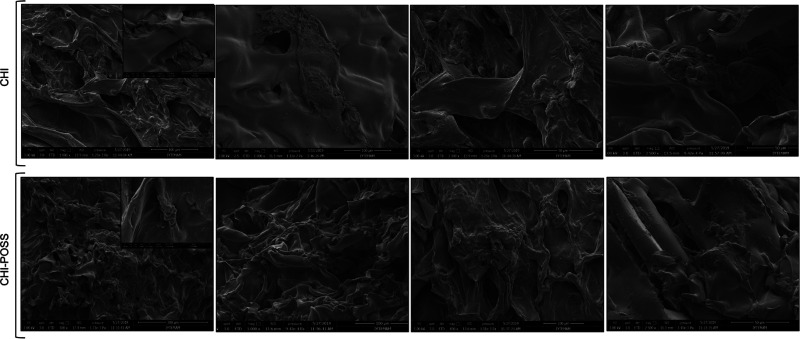
Scanning electron microscopy images of NIH/3T3 cells attached on CHI and CHI-POSS porous layers on the 3rd day of incubation with 500×, 1000×, and 2500× magnifications.

**Figure 16:**
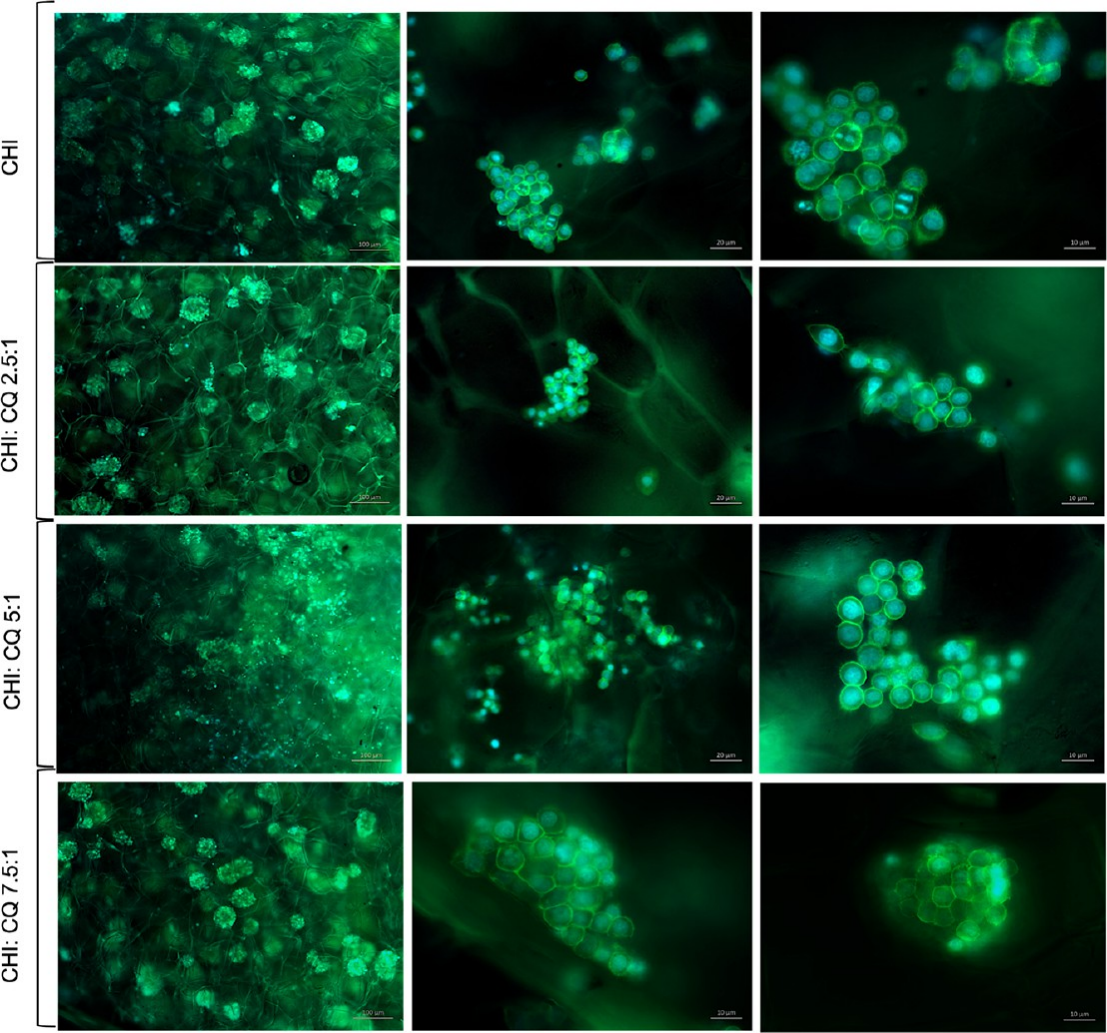
Fluorescence images of HS2 attachment and spreading on CHI-CQ nanofiber layers on the 7th day of incubation with 100, 20, and 10 μm scales.

**Figure 17:**
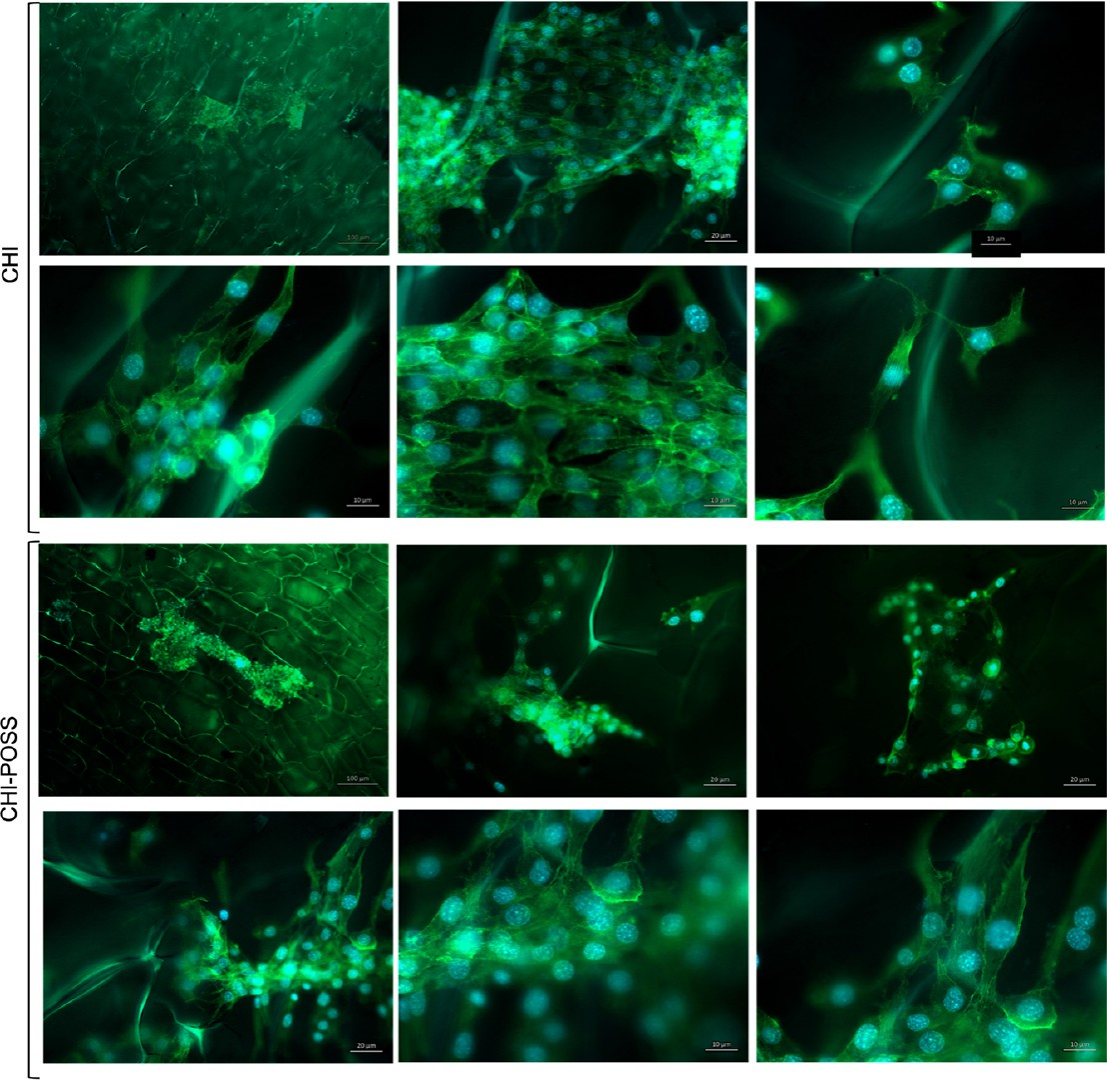
Fluorescence images of NIH/3T3 attachment and spreading on CHI and CHI-POSS porous layers on the 7th day of incubation with 100, 20, and 10 μm scales.

Cell attachment and spreading of NIH/3T3 and HS2 cells on each layer surface were investigated with SEM analysis at 3 days and fluorescence staining at 7 days of incubation. SEM images indicated that HS2 cells attached on the nanofiber layer with 3D morphology and started to form clusters (Figure 14). NIH/3T3 cells attached and spread on the chitosan porous layer with mostly elongated morphology, whereas on the CHI-POSS layer surface, fibroblasts attached and spread with 3D morphology, forming clusters (Figure 15). These alterations in cell morphology may arise from the change in pore size and 3D structure of the chitosan matrix with the addition of POSS nanoparticles. On the 7th day of incubation, fluorescence images showed that HS2 cells formed clusters with cell-to-cell interaction and located on nanofiber layers homogeneously. HS2 cells exhibited their characteristic morphology on nanofiber layers (Figure 16). NIH/3T3 cells also highly proliferated on CHI and CHI-POSS porous layers, showing their characteristic morphology. NIH/3T3 cells attached and proliferated in close contact with each other and on pore wall surfaces, elongating with their cytoskeleton (Figure 17).

#### 3.11.4 Cell Proliferation

**Figure 18:**
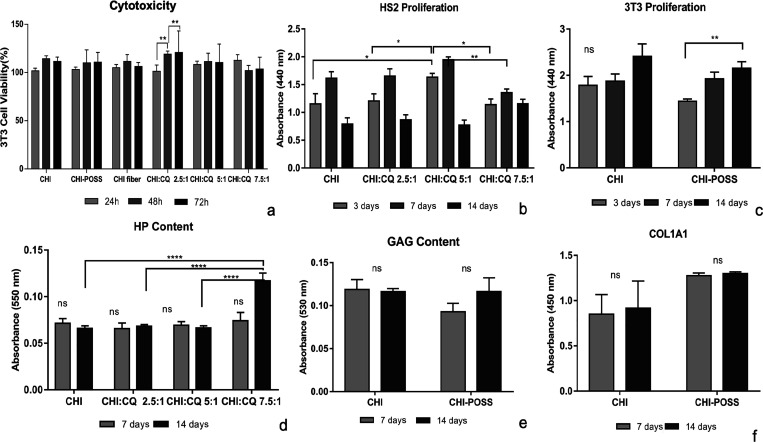
*In vitro* bioactivity results of NIH/3T3 and HS2 cell lines on fiber and porous layers: cytotoxicity (a); HS2 proliferation (b); NIH/3T3 proliferation (c); HP secretion (d); GAG secretion (e); Col1A1 secretion (f).

HS2 and NIH/3T3 proliferation at each layer of bilayer wound dressings was investigated with the WST-1 cell viability kit. Cells were cultivated with a density of 10^6^ cells/mL and incubated on each layer (1 × 1 cm) for 14 days. Cell viability results showed that NIH/3T3 fibroblasts proliferated on the CHI-POSS porous layer with an increasing trend during the incubation period. HS2 cells proliferated on CHI-CQ nanofiber layers up to 7 days. However, on the 14th day of incubation, it is considered that cell proliferation decreased due to the limited cultivation area for highly proliferated HS2 cells. At 3 days of incubation, HS2 cell proliferation was found to be significantly higher on the 5:1 CHI:CQ nanofiber layer when compared to control group chitosan (Figure 18b). NIH/3T3 cells proliferated on both CHI and CHI-POSS porous layers. However, fibroblast proliferation on the CHI-POSS composite layer gradually increased with incubation time. Hybrid POSS nanoparticles composed of a Si-based nanocage and tetramethylammonium organic groups showed a positive effect on fibroblast proliferation (Figure 18c). Similarly, Quignard et al. investigated the effect of nanosilica particles on the *in vitro* wound healing activity of skin fibroblast cells. *In vitro* results indicated that positively charged amine-functionalized nanosilica particles at appropriate concentrations of silicic acid were released as the bioactive silica form and promoted wound healing activity significantly by showing a positive impact on skin fibroblast proliferation.^9^

#### 3.11.5 HP, GAG, and Col1A1 Secretion

Hydroxyproline (HP) is a significant non-essential amino acid that is found in the skin and connective collagen and participates in extracellular matrix formation in wound healing, which can induce fibroblast migration in the injured wound area.^45,46,49^ HP secretion of HS2 and NIH/3T3 cells on bilayer sponges was evaluated with colorimetric assay for 7 and 14 days of incubation. Results indicated that similar HP content was observed for all groups at 7 days, whereas HP content on the CHI:CQ 7.5:1 group was found to be significantly higher at 14 days of incubation (Figure 18d).

GAGs are unbranched polysaccharide chains known as major constituents of the tissue extracellular matrix (ECM) with significant physiological functions such as providing structural and biochemical support to the embedded and surrounding cells. Among them, hyaluronic acid (HA) and chondroitin sulfates are major types of GAGs in skin tissue. The epidermis and dermis layers of skin tissue contain various GAGs as only 0.1–0.3% of the total skin weight. However, GAGs play a crucial role in skin volume and elasticity because of their large water-retaining capability. Dermal HA is mainly produced by skin fibroblasts. In addition to this, fibroblast physiology is also modulated by this GAG production as cell migration, proliferation, and cytokine production.^46,50,51^ GAG production of fibroblast cells on the porous layer was determined for 14 days. GAG production on the CHI layer was found to be higher on the 7th day due to the possible degradation byproducts of the neat chitosan matrix, which shows structural similarity with glycosaminoglycans. However, POSS incorporation leads to stability in the chitosan matrix by covalent and noncovalent interactions of its organic groups. Results also indicated that fibroblasts gradually produced GAG on the CHI-POSS nanocomposite layer at the end of the incubation period (Figure 18e).

In skin tissue, the epidermis layer is tightly connected to the extracellular matrix (ECM) of the dermis, which is mainly composed of collagen fibers synthesized by the dermal fibroblast cells. Dermis is composed of two main layers as the upper region (papillary dermis) consisting of a high amount of type III collagen and the lower region (reticular dermis) containing type I collagen at high levels. The wound healing process can be divided into four stages of hemostasis, inflammation, proliferation, and remodeling. In the proliferative phase, fibroblasts synthesize and organize collagen and other ECM components to generate new tissues.^52^ In this study, collagen deposition of fibroblast cells on the CHI-POSS nanocomposite layer was investigated for 14 days of incubation. Higher collagen deposition was obtained for both 7th and 14th days on the CHI-POSS porous layer. Results indicated that POSS incorporation enhanced collagen secretion of fibroblasts cultivated on the porous layer (Figure 18f). In the literature, Park and co-workers used a pig model to conduct *in vivo* experiments to investigate the function of Si in the wound microenvironment. Si in the CTS-Si membrane enhanced biological responses for fibroblast proliferation in addition to showing increased collagen deposition densities.^12^

## 4 Conclusions

*C. quadrangularis* as bioactive herbal extract is specially used in tissue regeneration due to its bioactive constituents such as anabolic steroidal substances, phytosterols, β-sitosterol, ascorbic acid, carotene, vitamin C, and inorganics including potassium, calcium, zinc, sodium, iron, lead, cadmium, copper, calcium oxalate, and magnesium. However, the direct use of bioactive extracts may cause some toxic effects on the wound area. Thus, encapsulation of these bioactive agents in the polymer matrix prevents possible toxic effects as well as provides controlled or sustained release at the wound site over time. Thus, in this study, it is aimed to design bioactive bilayer wound dressing to mimic the dermal layers as well as to obtain wound healing activity with sustained release of *C. quadrangularis* extract. Fiber and sponge layers were properly integrated throughout the fabrication process. Results showed that the initial burst release of CQ extract was observed at 6 h, whereas the sustained release of CQ was obtained up to 4 days. Water vapor permeability values of bilayer sponges were found in the appropriate range for wound dressing applications. Bilayer sponges did not show any toxic effect on fibroblast cells. In addition, fibroblast and keratinocyte cells attached on each layer and proliferated successfully. *In vitro* wound healing assay indicated that CQ extract loading induced both cell migration and proliferation at the wound area. In addition, POSS incorporation also induced the collagen production of fibroblast cells. As a result, bilayer sponges loaded with natural CQ extract showed promising effects as a potential biomaterial for wound healing applications.
